# Truth of dare: The principles of storytelling in Ophthalmology services - review

**DOI:** 10.22336/rjo.2021.43

**Published:** 2021

**Authors:** Consuela-Mădălina Gheorghe, Victor Lorin Purcărea, Iuliana-Raluca Gheorghe

**Affiliations:** * Department of Marketing and Medical Technology, “Carol Davila” University of Medicine and Pharmacy, Bucharest, Romania

**Keywords:** principles of storytelling, ophthalmology services, digital storytelling

## Abstract

Storytelling has been long researched in literature and its benefits have been acknowledged. Recently, storytelling has widely been used in health care and has proved to be very helpful in health education. However, the impact of storytelling in several medical specialties has not been investigated. The aim of this paper was to highlight the benefits of using storytelling in Ophthalmology.

As technology evolved, storytelling has been transformed into digital storytelling with more benefits for both health care consumers and health care providers. Apart from promoting a constant health education, digital storytelling offers the opportunity to raise the health care consumers’ awareness on a specific ophthalmologic disease, and identify proper methods to prevent it, as well as identify the risk factors accordingly.

## Introduction

Nowadays, storytelling has been used and considered a powerful strategic tool of Marketing Communication, facilitating the transfer of ideas in a harmonious and consistent way, to stand out from other similar services, while building, at the same time, a positive reputation for the organization. In essence, storytelling is a competitive advantage for any organization, especially for health care organizations [**1**]. On the other hand, the use of storytelling for health care consumers may work as an environment for sharing experiences and for the creation of supporting groupings, such as Patient Online Communities [**2**].

It is interesting to emphasize that in health care, when most of the times, specialists become obsessive about evidence-based cues, the power of storytelling in health care education is being promoted [**3**]. Moreover, storytelling, as a means of communicating health messages, is cost effective, it is not dependable on the literacy of the target population, does not require some sophisticated equipment or access to a reliable energy supply, but it is necessary for the sender of the message, the storyteller, to understand the cognitive structures of the target audience and to embed them accordingly [**4**]. 

As health education is eclectic and rapidly evolving, health experts put a lot of emphasis on both identifying evidence-based interventions, as well as, disseminating them widely [**5**]. Along with this change, a new type of storytelling emerged - digital storytelling. Early research that focused on the investigation of digital storytelling in health care education revealed that many individuals have commented on the personal nature of the stories and that these experiences brought to life the theoretical knowledge they had of the health and illness in an effective, affective, and reflective manner [**6**]. However, the application of storytelling principles in different medical specialties is still under discussion. Thus, the aim of this paper was to highlight the benefits of using storytelling in Ophthalmology. 

## What is storytelling?

Storytelling has been part of the human culture for thousands of years and it is a powerful and an enduring means of interpersonal communication [**7**]. Storytelling is an oral communication, structured around a logical sequence of events, utilizing human and/ or animal characteristics with personalities and emotional qualities, presented with voice, gesture and facial expression [**8**]. Similarly, storytelling presents and organizes consecutive facts in a logical and coherent way, beginning with the introduction of the events (stories) and ending with the solving of the existing problem [**9**]. As such, Kakroo defined a story as an umbrella concept that consists of facts with emotional elements in order to trigger certain behaviors in individuals [**10**]. Yang found that by triggering emotional reactions, the stories become more persuasive than statements or quantitative information [**11**]. The phenomenon in the story was narrowly regarded as the story per se, while the process to describe the phenomenon was named narrative. 

Further, Kakroo emphasized that a story has three elements [**10**]:

- the plot contains the beginning, interval, and the ending, describing the actions in a story;

- the characters are the persons who act in the story;

- the aesthetics are the costumes, the decoration, the telling styles, and the rhetorical skills of the storyteller. 

The way interactions are established between the characters of the story triggers the attractiveness of the message for the recipients. The story per se enables the transfer of complex emotions due to its structure. Moreover, the emotional part of the story becomes effective because the content stored in the human consciousness is associated with a series of short stories in a dynamic process [**12**].

Research on the effectiveness of storytelling shows that its use impacted communication and behavior, particularly the following [**13**,**14**]:

- raises the awareness on a specific topic, theme, or advertisement by providing facts or direct descriptions of the features and benefits of the service;

- perception on the quality - the narrative builds a more positive perception on the quality of services;

- builds an attitude - the narrative builds more positive attitudes;

- willingness to act - the narrative builds higher behavioral impacts;

- engagement in the story - the narrative builds a higher commitment to its content by the people who listen. 

According to Znanewitz and Gilch, the following criteria are used to create effective storytelling [**15**]:

- the right story is based on time facts such as own experiences or evidence-based experiences;

- entertainment and excitement - aligned with up-to-date trends that are relevant to the target group;

- uniqueness - the emotional elements embedded should be different from the stories of other organizations;

- conciseness - the story or the facts may be summarized in a few sentences;

- simplicity - the story is simple, not too complex. Stringent plot, sparse details, letting the recipient be a co-creator;

- connectivity - gaps, open ends, incomplete background information, to let the recipient become a co-creator and imagine them;

- brand persona - used known celebrities or archetypes for quick and easy connection with the audience. 

Due to its usefulness, the storytelling approach is increasingly becoming a core element in the influential process in several fields such as education, teaching, leadership, culture, history, marketing and on a whole range of other functionalities across the multitude of human activities [**16**].

## Storytelling in health care

Patient storytelling can serve as educational instruments for both health care providers and other health care consumers.

Usually, health education addresses topics related to disease prevention and promotion, detection of illness, rehabilitation, and long-term treatment in a continuum manner. Simonds defined health education as aimed at bringing about behavioral changes in individuals, groups and larger populations from behaviors that are presumed to be detrimental to health, to behaviors that are conducive to present and future health [**17**]. In the same vein, Green characterized health education as any combination of learning experiences designed to facilitate voluntary adaptations of behavior conducive to health [**18**]. Health education is influenced by five levels of factors that relate to the individual, interpersonal elements, institutional, community and public-policies. 

Patient stories are different from medical history that health care providers are used to hearing [**19**]. It has been concluded that they offer a valuable contribution to the vast number of information resources intended to ease the change and support the transformation in health services [**20**].

As patient storytelling can become very useful for other peers by revealing the medical experience in an accurate and emotional manner, it also offers many benefits to the health care providers by understanding the entire experience of the patients, not only the medical experience [**19**]. Consequently, patient storytelling can lead to improved collaboration and communication between patients and physicians, leading, in fact, to an improved patient-centered care. Moreover, patient storytelling may close the gap between the patient and the health care provider’s experiences by offering valuable clinical information beyond the scope of practice, making them understand where change is necessary [**21**]. In addition, many medical providers stated that patient stories offer them unique opportunities and insights into some perspectives they would not be aware of [**21**]. However, in these troubled times, physicians do not have the required time to ensure an active listening process, but rather a monitoring process. More exactly, on one hand, patients feel the need to become co-producers when their health is at stake and express their desire to become more empowered with information [**22**], and on the other hand, physicians suffer frequently from burnout and do not have the time to give special attention to all their patients [**23**].

In this context, new technology offered the opportunity to empower patients and health care staff by updating the conventional storytelling with digital storytelling, shaped by short videos consisting of dynamic images, music, and voice-over [**24**].

The stories are told by real people about real experiences, and have the following essential characteristics [**25**]:

- brief: short digital stories;

- simple: the images, voice-over or the music are carefully selected without needing complex technology and equipment;

- personal: revealing important personal health issues that involve the storyteller;

- about the story: a way of developing associated skills;

- respectful of others’ feeling and experiences;

- created in a spirit of collaboration and partnership. 

Moreover, the principles of a good digital story relate to the following [**25**]:

- have a purpose;

- are descriptive;

- are interesting, captivating, and something the listener or the reader can relate to;

- are coherent;

- make a point;

- inspire listeners or readers to reflect and act or change their behavior.

From a health care consumer perspective, the digital storytelling touches hearts and influences minds, providing opportunities for reflection [**26**] and offers collaborative learning with the potential of promoting greater understanding between patients. 

From a physician perspective, the benefits of storytelling involve [**20**]:

- development of the skills required to follow a narrative thread, tolerating ambiguity, and surrendering to the story;

- the adoption of multiple and contradictory points of view, however, finding the right balance of information;

- an ability to enter the storyteller’s reality and to understand how he/ she perceives his/ her reality and the facts;

- to gain insight into the use of image and metaphor;

- to acknowledge the use of imagination in being transported to the storyteller’s reality. 

Through digital storytelling, patients or service users provide rich sources of health-related stories that should influence and change the clinical practice and the experience of other health care consumers in an evolving community cohesion. 

## Digital storytelling in Ophthalmology

Digital storytelling in Ophthalmology is generally used as in any other medical specialty. However, the American Academy of Ophthalmology uses digital storytelling not only to provide health education, but also to embed the unexpected element in their message, followed by emotional and credible facts. 

For instance, titles such as “Vision rehabilitation revitalized 103-year-old woman”, “persistence saves burn survivor’s eye”, “cutting-edge transplant restores veteran’s sight”, “news reporter rescued from severe dry eye” or “cornea transplant restores young boy’s sight after fishing incident” are the proof of applying the principles of storytelling in an efficient manner. 

The digital story entitled “Glaucoma surgery saves woman from a life in darkness” [**27**] tells the story of Peggy Wellman who had trouble seeing and who was diagnosed by the ophthalmologist Chasidy Singleton, to have glaucoma, and was told that she was going blind. The story has a plot, two characters and emotional facts supported by a few photos. The story emphasized the stages of glaucoma and the treatment, as well as the story of Peggy with a happy ending due to a surgery conducted at the right moment. Apart from the main plot, the story also includes some additional information about the disease and what are the risk factors, in other words, building awareness and health educational information. At the same time, the unexpected element was embedded in the message, when the user reads that glaucoma can be treated with eyedrops or an in-office laser treatment, when diagnosed early, or, despite being diagnosed rather late, you can still save your eyesight by selecting a surgical procedure, even if it will not save the eyesight completely. 

## Conclusion

Storytelling is a means of communicating a message, using emotional elements in the message. Storytelling is told by a real storyteller, and the story is a real experience. In health care, storytelling is employed with the aim of assessing health education. However, in the present context, storytelling in health care has evolved to digital storytelling. The Ophthalmology digital storytelling has the scope of educating the potential health care consumers and of raising their awareness on a specific ophthalmologic disease, helping them identify the proper methods to prevent and diagnose the disease and the associated risk factors. Moreover, in order to attract their attention, they embed an unexpected element in the message.

**Conflict of Interest statement**

The authors state no conflict of interest.

**Acknowledgements**

None.

**Sources of Funding**

None.

**Disclosures**

None.

## References

[1] Gheorghe CM, Purcarea VL, Gheorghe IR (2018). A marketing perspective on consumer perceived competition in private ophthalmology services. Romanian Journal of Ophthalmology.

[2] Gheorghe CM, Purcarea VL, Gheorghe IR (2018). Don’t cry over spilt milk: a review on consumer-based corporate reputation in private ophthalmology services. Romanian Journal of Ophthalmology.

[3] Haigh C, Hardy P (2011). Tell me a story- a conceptual exploration of storytelling in healthcare education. Nurse Education Today.

[4] Silver D (2001). Songs and storytelling: bringing health messages to life in Uganda. Education for Health.

[5] Rimer BK, Glanz K, Rasband G (2001). Searching for evidence about health education and health behaviour interventions. Health Education and Behavior.

[6] Sumner T (2009). About Patient Voices.

[7] Zatwarnicka-Madura B, Nowcki R (2018). Storytelling and its impact on effectiveness of advertising. ICOM Conference, 8th International Conference on Management.

[8] Greene H, Koh K, Bonnici J, Chase J (2015). The value of storytelling in the Marketing curriculum. Journal of the Academy of Business Education.

[9] Labov W (1997). Some further steps in narrative analysis. Journal of Narrative and Life history.

[10] Kakroo U (2015). 5 ways to use storytelling in your Social Media Marketing.

[11] Yang C (2013). Telling tales at work: an evolutionary explanation. Business Communication Quarterly.

[12] Hong Kun C, Byueong-Hyun M, Jae Young K (2019). Analysis on consumer behaviour model for storytelling advertising of high-tech products: focusing on identification and empathic response. International Journal of Applied Business and Economic Research.

[13] Adval R, Wyer RS (1998). The role of narratives in consumer information processing. Journal of Consumer Psychology.

[14] Matilla AS (2000). The role of narratives in the advertising of experiential services. Journal of Service Research.

[15] Znanewitz J, Gilch K (2016). Storytelling - a guideline and an application in the Bundeswehr’s (personnel) marketing. Transfer: Werbeforchungs and Praxis.

[16] Kadembo E (2012). Anchored in the story: the core of human understanding, branding, education, socialization and the shaping of values. The Marketing Review.

[17] Simonds S (1976). Health Education in the mid-1970s: state of the art. In Preventive Medicine.

[18] Green LW, Krenter MW (2005). Health Promotion Planning: an educational and ecological approach.

[19] Ashdown LC, Maniate JM (2020). Determining patient readiness to share their health care stories: a tool for prospective patient storytellers to determine their readiness to discuss their healthcare experiences. Journal of Patient experience.

[20] Charon R (2006). Narrative Medicine: Honoring the stories of illness.

[21] Milne V, Buchanan F, Tepper J, Petch J (2017). How patient stories are re-shaping health care.

[22] Gheorghe IR, Gheorghe CM, Purcarea VL (2018). Measuring the perceived quality of ophthalmology services in private organizations. A marketing perspective. Romanian Journal of Ophthalmology.

[23] Popa-Velea O, Diaconescu LV, Gheorghe IR, Olariu O, Panaitiu I, Cernițanu M, Goma L, Nicov I, Spinei L (2019). Factors associated with burnout in medical academia an exploratory analysis of Romanian and Moldavian physicians. International Journal of Environmental Research and Public Health.

[24] Gray JAM (2002). The resourceful patient.

[25] Heath C, Heath D (2007). Made to stick: why some ideas survive and others die.

[26] McDrury J, Alterio M (2002). Learning through storytelling in higher education: using reflection and experience to improve learning.

[27] American Academy of Ophthalmology Glaucoma surgery saves woman from a life in darkness.

